# Age-Related Regeneration of Osteochondral and Tibial Defects by a Fibrin-Based Construct *in vivo*

**DOI:** 10.3389/fbioe.2020.00404

**Published:** 2020-05-05

**Authors:** Xue Feng, Peifang Xu, Tao Shen, Yihan Zhang, Juan Ye, Changyou Gao

**Affiliations:** ^1^MOE Key Laboratory of Macromolecular Synthesis and Functionalization, Department of Polymer Science and Engineering, Zhejiang University, Hangzhou, China; ^2^Department of Ophthalmology, The Second Affiliated Hospital of Zhejiang University, School of Medicine, Hangzhou, China

**Keywords:** fibrin-based scaffold, bone marrow-derived mesenchymal stem cells, age-related tissue regeneration, adaptive biomaterials, tissue microenvironment

## Abstract

Tissue–biomaterial interactions in different microenvironments influence significantly the repair and regeneration outcomes when a scaffold or construct is implanted. In order to elucidate this issue, a fibrin gel filled macroporous fibrin scaffold (fibrin-based scaffold) was fabricated by loading fibrinogen via a negative pressure method, following with thrombin crosslinking. The macroporous fibrin scaffold exhibited a porous structure with porosity of (88.1 ± 1.3)%, and achieved a modulus of 19.8 ± 0.4 kPa at a wet state after fibrin gel filling, providing a suitable microenvironment for bone marrow-derived mesenchymal stem cells (BMSCs). The *in vitro* cellular culture revealed that the fibrin-based scaffold could support the adhesion, spreading, and proliferation of BMSCs in appropriate cell encapsulation concentrations. The fibrin-based scaffolds were then combined with BMSCs and lipofectamine/plasmid deoxyribonucleic acid (DNA) encoding mouse-transforming growth factor β1 (pDNA-TGF-β1) complexes to obtain the fibrin-based constructs, which were implanted into osteochondral and tibial defects at young adult rabbits (3 months old) and aged adult rabbits (12 months old) to evaluate their respective repair effects. Partial repair of osteochondral defects and perfect restoration of tibial defects were realized at 18 weeks post-surgery for the young adult rabbits, whereas only partial repair of subchondral bone and tibial bone defects were found at the same time for the aged adult rabbits, confirming the adaptability of the fibrin-based constructs to the different tissue microenvironments by tissue-biomaterial interplays.

## Graphical Abstract

A fibrin-based construct was fabricated to investigate its repair effects in different tissue microenvironments, namely, osteochondral tissue and tibia in young adult rabbits and aged adult rabbits. Influenced by the interactions between the implanted fibrin-based construct and respective tissue microenvironment, the osteochondral defects and tibial defects were partially or completely repaired, confirming the adaptability of the construct to different tissue microenvironments.

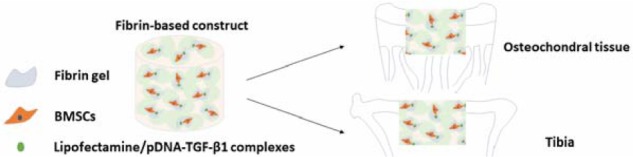

## Introduction

The damaged tissues need to be treated properly and timely to avoid further degeneration and even tissue dysfunction, especially for those with limited self-regenerative capability such as cartilage (Goldring and Goldring, 2016). The damage of cartilage and bone is mainly caused by trauma during competitive sports and traffic accidents as well as aging and osteoarthritis, resulting in long-term organ deterioration and thereby influencing human health and life quality greatly (Puppi et al., 2010; Nukavarapu and Dorcemus, 2013; Makris et al., 2015). So far various clinical or surgical methods such as arthroscopic debridement, abrasion arthroplasty, chondroplasty microfracture, mosaicplasty or autograft, allograft, autologous chondrocyte transplantation (ACI), and matrix-assisted ACI (MACI) have been applied to treat cartilage and osteochondral defects of different degrees (Panseri et al., 2012; Nukavarapu and Dorcemus, 2013). In contrast, owing to its spontaneous self-regeneration, the bone with a defect smaller than a critical size (two times of bone diameter of long bones; Bigham-Sadegh and Oryan, 2015) could be self-repaired or regenerated with traditional therapies such as Ilizarov method, despite that large bone defects still need to be treated with autologous/allogenic transplantation and synthetic bone substitutes, usually consisting of metal or calcium phosphate and bioactive glasses (Puppi et al., 2010; Huey et al., 2012). Nonetheless, some disadvantages exist when using the above-mentioned therapies, such as fibrocartilage formation, tissue degeneration in long-term observation, limited donor supply, donor site degeneration, imperfect fusion or integration of autografts or allografts within defects, possibly immune rejections, disease transmission, and so on (Correa and Lietman, 2017). Tissue engineering applies “biological, chemical, and engineering principles toward the repair, restoration, or regeneration of living tissue by using biomaterials, cells, and factors alone or in combination” (Swieszkowski et al., 2007), which provides promising alternatives for dealing with chondral or bone defects.

Interactions between biomaterials and native tissue microenvironments exert important roles during remodeling and regeneration of damaged tissues or organs by transplantation of tissue engineering scaffolds. Not only the spatial or regional diversity but also age-related changes should be involved when elaborating differences in tissue microenvironments. In elderly individuals, there is a chronic, low-graded inflammation featured by a two- to fourfold increase in serum levels of pro-inflammatory cytokines such as tumor necrosis factor (TNF)-α, interleukin (IL)-6, and matrix metalloproteinase (MMP)-3 in comparison to young individuals. These pro-inflammatory cytokines may further cause the functional decline of the adaptive immune system, and production of reactive oxygen species (ROS) that impair deoxyribonucleic acid (DNA) and cell membrane via lipid peroxidation, and functional cellular and extracellular proteins, and degrade tissue environment, leading to more susceptible to degenerative diseases such as osteoarthritis and osteoporosis (Khansari et al., 2009; Cevenini et al., 2010; Freund et al., 2010). Besides, chronic inflammation could also contribute to disruption of stem cells via either directly driving stem cell differentiation by inflammatory mediators or indirectly destroying stem cell niches with the aid of secreted proteases and destructive activities by immune cells (Freund et al., 2010). It is reported that mesenchymal stem cells (MSCs) from younger murine show better adhesion and proliferation behaviors and have better chondrogenic and osteogenic potential than those from older murine (Kretlow et al., 2008). It is also documented that transplantation of MSCs derived from younger donors into old mice significantly slows the loss of bone density and prolongs the life of old mice in treatment of age-related osteoporosis (Shen et al., 2011). Besides, senescent cells are accumulated with age, which go through dramatic changes in gene expression, metabolism, and epigenome (de Magalhaes and Passos, 2018). They also exhibit a distinct senescence-associated secretory phenotype (SASP) including pro-inflammatory cytokines, chemokines, growth factors, and proteins that degrades extracellular matrix, and thereby significantly alters tissue structure and the local microenvironment, inducing aging and age-related pathology despite of their suppression of the cancer development in early life (Campisi et al., 2011; de Magalhaes and Passos, 2018). Therefore, the organisms show an age-related decline in tissue functions and remodeling capacities as a result of differences in tissue environment and resultant negative effects.

During the processes of biomaterial–tissue environment interactions, a series of spatiotemporal changes of biomaterials’ structures and properties occur under the stimulation of physiological and pathological microenvironments, which in turn alters the host microenvironments and further influence the host responses to the transplanted biomaterials (Dai et al., 2019). In this regard, the simultaneous regeneration of cartilage and subchondral bone has been achieved by using a homogeneous construct consisting of a poly(lactide-co-glycolide) (PLGA) scaffold filled with fibrin gel, bone marrow-derived MSCs (BMSCs), and lipofectamine/plasmid DNA encoding transforming growth factor (TGF)-β1 (pDNA-TGF-β1) complexes (Wang et al., 2010a; Li et al., 2014; Dai et al., 2019). This construct thus shows the unique adaptive ability to the microenvironments of different tissues or organs, which achieves the regeneration of ear cartilage and eyelid tarsal plate too but results in fibrous tissue only when implanted subcutaneously (Wang et al., 2010a; Li et al., 2014; Dai et al., 2019). The porous PLGA scaffold serves as a spatiotemporal support before neo-tissue ingrowth, and the fibrin gel can deliver cell and genes, and provide appropriate microenvironments for cells (Whelan et al., 2014; Dai et al., 2019). TGF-β1 is a latent polypeptide *in vivo* and participates in tissue regeneration processes after ligand activation (Xu et al., 2018). BMSCs have paracrine functions besides their multi-differentiation ability influenced by the tissue microenvironments, leading to the regeneration of different types of tissues by interacting with the host tissues (Reagan and Rosen, 2016). Fricain et al. (2013) achieved ectopic osteogenesis subcutaneously in mice and intramuscularly in goat, and *in situ* bone formation at the femoral condyle defect of rat, transversal mandibular, and tibia in goat by transplantation of a nano-hydroxyapatite-pullulan/dextran polysaccharide composite macroporous scaffold, coinciding with the views of self-adaptability of biomaterials. Nonetheless, in all these studies, the age-related changes in tissue microenvironments as well as their interactions with implanted biomaterials during regeneration have not been considered. Moreover, the acidic degradation products of PLGA are believed unfavorable to cartilage regeneration to some extent due to the aseptic inflammatory responses (Chen and Liu, 2016).

**FIGURE 1:**
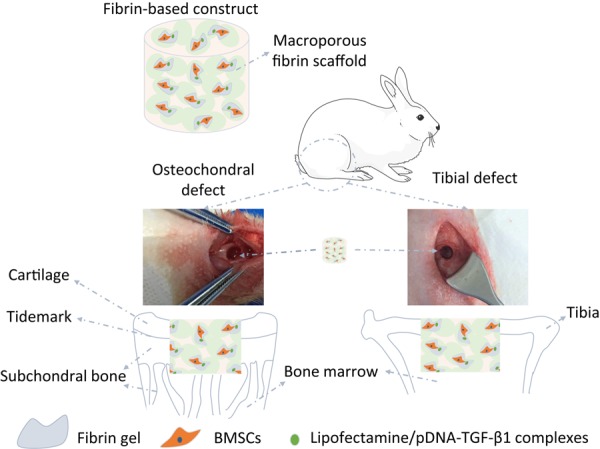
Illustration of the structure and implantation of the fibrin-based construct incorporated with bone marrow-derived mesenchymal stem cells (BMSCs) and lipofectamine/plasmid DNA encoding mouse-transforming growth factor β1 (pDNA-TGF-β1) complexes into osteochondral defects and tibial defects *in vivo*.

Fibrinogen exists abundantly in serum, and can be easily gelated to form hydrogels (Tajdaran et al., 2015; Ricles et al., 2016; Heher et al., 2018). It can also be manufactured into macroporous scaffold with a significantly improved strength compared with fibrin gel (Snyder et al., 2014; Dai et al., 2016). In this study, a fibrin-based construct is developed by combing the advantages of fibrin scaffold and hydrogel, aiming at evaluating the repair efficacies of this construct on the osteochondral and tibial defects at young and aged adult rabbits (Figure 1), and exploring the spatial-associated and age-related differences in tissue microenvironments as well as their interplays with biomaterials. This fibrin-based construct consists of a macroporous fibrin scaffold and fibrin gel within the macroporous scaffold as a reservoir for BMSCs and lipofectamine/pDNA-TGF-β1 complexes (Whelan et al., 2014; Song et al., 2018). On this basis, we attempt to investigate the adaptability of this fibrin-based construct to different microenvironments in both spatial-associated and age-related manners.

## Experimental Section

### Reagents and Materials

Polycaprolactone (PCL, average Mn 80 kDa) from Sigma–Aldrich (United States) and poly(vinyl alcohol) (PVA, 86–89% hydrolyzed, low molecular weight) from Alfa Aesar (United States) were used to prepare PCL microspheres as porogen for fabricating fibrin macroporous scaffold by using fibrinogen (Boya Bio-pharmaceutical, China) and thrombin from bovine (EMD Millipore Corp., United States). Lipofectamine 2000 obtained from Invitrogen (United States) was used as a vector of pDNA-TGF-β1. Fetal bovine serum (FBS) and Dulbecco’s modified Eagle’s medium (DMEM) were obtained from Gibco (United States) for cell culture *in vitro*. Ultrapure water was prepared by a Milli-Q^®^ ultrapure water purification system (Merck, United States). Other reagents were used as received without further purification.

### Fabrication of Fibrin-Based Scaffold

The fibrin-based scaffold represents a macroporous fibrin scaffold filled with fibrin gel. The macroporous fibrin scaffold was prepared by a porogen-leaching method using PCL microspheres as the porogen (Dai et al., 2016). Briefly, PCL microspheres were fabricated by a single emulsion method and separated by standard sieves to collect those with diameters of 100–200 μm, which were then mixed with fibrinogen at a mass ratio of 1.5: 1 according to the optimal mixing ratio determined in our previous work (Dai et al., 2016). The mixture was transferred into a polyethylene (PE) mold, compressed to release air to ensure close contact of the two components, and subsequently placed at 65°C for 1 h. In this heating process, PCL microspheres were partially melted and interconnected with each other. After cooled to room temperature, the fibrinogen component was crosslinked by thrombin at 37°C for 24 h, followed by removal of PCL with acetone. The macroporous fibrin scaffold was rinsed with graded ethanol solutions to remove residual acetone, and finally lyophilized. Fibrinogen solution with a concentration of 20 mg/mL was introduced into the pores of the macroporous scaffold under negative pressure, and then crosslinked by thrombin at 37°C for 30 min to obtain the fibrin-based scaffold.

### Characterization of the Fibrin-Based Scaffold

The morphology of PCL microspheres, macroporous fibrin scaffold, and fibrin-based scaffold was observed by a field emission scanning electron microscope (FESEM; S4800, Hitachi, Japan). The diameters of 200 PCL microspheres were measured using Image J software (National Institutes of Health, United States), and Gaussian distribution formula was used to fit their diameter distribution and average diameter.

The porosity (P) of the macroporous fibrin scaffold was calculated according to the following equation, where *V*_0_ and *W*_0_ represent the volume and mass of the macroporous fibrin scaffold, and ρ represents the density of fibrin (assumed as 1.2 g/mL).

$$P=100\%\times \left(V_{0}-W_{0}/\rho \right)/V_{0}$$

The stress–strain curves of the macroporous fibrin scaffold and the fibrin-based scaffold in the dry and wet states were measured by a universal mechanical testing instrument (5543A, Instron, United States) with a compression rate of 0.5 mm/min. The measurement in the wet state was performed after the scaffolds were completely hydrated in 0.01 M phosphate buffered saline (PBS). The compressive modulus was calculated according to the slope of the initial linear part of the stress–strain curves.

The weight loss of the macroporous fibrin scaffold and fibrin-based scaffold was measured in 0.01 M PBS at 37°C for 21 days. The relative weight was calculated according to 100 × *W*_*t*_/*W*_0_ (%), where *W*_*t*_ represents the mass at time t.

### Isolation and Culture of BMSCs

BMSCs were harvested from tibial bone marrow from male New Zealand white (NZW) rabbits following the procedures described previously (Dai et al., 2016), which were cultured to Passage 2 and used before Passage 4. The procedures were carried out in accordance with the Guidelines for Care and Use of Laboratory Animals of Hangzhou Medical College and approved by the Animal Ethics Committee of Hangzhou Medical College (approval ID: 20200032). Briefly, after anesthesia and disinfection with iodophor, the rabbit was punctured from tibia through a 12-gauge needle, and 2–3 mL bone marrow was aspirated into a 5 mL syringe containing 5000 U heparin. The obtained bone marrow was diluted with a complete DMEM medium containing 10% v/v FBS, 100 μg/mL streptomycin, and 100 U/mL penicillin, followed by centrifugation at 2000 r/min for 10 min to remove heparin and fat. This process was repeated for two times. The cell precipitation was cultured in the medium at 37°C and 5% CO_2_. The BMSCs were routinely passaged after 80% confluency.

### *In vitro* Cytocompatibility Evaluation and Effects of Cell Concentration on the Behavior of the Fibrin-Based Scaffold

3-(4,5-Dimethyl-2-thiazolyl)-2,5-diphenyl-2-H-tetrazolium bromide (MTT) assay was used to evaluate the viability of BMSCs seeded within the fibrin-based scaffold. The macroporous fibrin scaffold with a diameter of 10 mm and thickness of 2 mm was sterilized with 75% ethanol for more than 4 h, and then exchanged with complete DMEM to remove ethanol. The excessive medium remaining in the scaffold was adsorbed with sterilized filter papers. BMSCs were digested, centrifuged, and re-suspended in sterilized fibrinogen solution (20 mg/mL) with a concentration of 5.0 × 10^6^/mL, which were then encapsulated into the macroporous fibrin scaffold under negative pressure and sterile condition. The fibrinogen solution/BMSCs filled macroporous scaffold was transferred into a 48-well culture plate, immersed in 25 U/mL thrombin and incubated at 37°C and 5% CO_2_ for 30 min to form the fibrin gel. 1 mL complete medium was added to each well, and the fibrin-based scaffold/BMSCs was cultured at 37°C and 5% CO_2_. The medium was changed every 3 days. At the time point of 1, 4, and 7 days, the medium was removed, and the fibrin-based scaffold/BMSCs was continually cultured in 1 mL complete medium containing 0.5 mg/mL MTT for 4 h. After the medium was removed, the BMSCs laden fibrin-based scaffold was cut into pieces, and 1 mL dimethyl sulfoxide was added into each well, followed by incubation at 37°C for 15 min and shaking for 10 min to assure complete dissolution of the formed formazan crystals. The mixture was centrifuged at 2000 r/min for 5 min, and the absorbance of the supernatant at 570 nm was recorded by a multiplate reader (Infinite M200PRO, Tecan, Switzerland).

The morphology of BMSCs within the fibrin-based scaffold was observed by a confocal laser scanning microscope (CLSM; LSM780, ZEISS, Germany). The fibrin-based scaffold/BMSCs was incubated at 37°C and 5% CO_2_ for 24 h before they were fixed with 4% w/v paraformaldehyde/PBS at 4°C overnight. After permeated in 0.1% w/v Triton X-100 at 4°C for 10 min and blocked in 1% w/v BSA at 37°C for 1 h, BMSCs were observed by CLSM after staining cell nuclear and cytoskeleton with 1 μg/mL 2-(4-amidinophenyl)-6-indolecarbamidine dihydrochloride (4’,6-diamidino-2-phenylindole) (DAPI; Sigma–Aldrich, United States) and 1 U/mL rhodamine-labeled phalloidin (Invitrogen, United States) at 37°C for 2 h, respectively.

The effects of cell concentration on the cellular viability and degradation behavior of the fibrin-based scaffold were also studied. After degradation, the relative weight was calculated according to 100 × *W*/*W*_0_ (%), where *W* and *W*_0_ represent the weight of the fibrin-based scaffold incubated for 7 days and the initial weight of the macroporous fibrin scaffold at dry state, respectively. Because of the low mass ratio (about 3%) of fibrin gel and BMSCs in the fibrin-based scaffold after freeze-drying, the weight of fibrin gel and BMSCs was neglected in order to assure aseptic operation.

### Extraction of pDNA-TGF-β1

pDNA-TGF-β1 purchased from China Jiliang University was propagated by growing Luria–Bertani medium containing 0.1 mg/mL ampenicillin for 12 h, and then extracted using Axyprep Maxi Plasmid Kit (Axygen Bioscience, United States) according to the manufacturer’s instructions (Li et al., 2014). The pDNA-TGF-β1 was used for the subsequent animal experiment *in vivo*.

### Fabrication of the Fibrin-Based Construct

The fibrin-based scaffold with a diameter of 4 mm and thickness of 4 mm was loaded with BMSCs/(lipofectamine/pDNA-TGF-β1) complexes to obtain the fibrin-based construct. The gene complexes were prepared by adding pDNA-TGF-β1 into lipofectamine 2000 at a mass to volume ratio of 1: 2 [m_pDNA–TGF–β 1_ (μg): V_lipofectamine 2000_ (μL)] and incubating the mixture at 37°C for 25 min to assure sufficient complexation of the two components. The complexes were subsequently mixed with fibrinogen solution, achieving a final concentration of 200 μg/mL (pDNA-TGF-β1) and 20 mg/mL (fibrinogen), respectively. The detached BMSCs were re-suspended in this solution with a concentration of 1.5 × 10^6^ cells/mL, which was then loaded into the macroporous fibrin scaffold as aforementioned. This construct was used for the following animal experiment.

### Animal Experiment

All animal procedures were performed in accordance with the Guidelines for Care and Use of Laboratory Animals of Hangzhou Medical College and approved by the Animal Ethics Committee of Hangzhou Medical College (approval ID: 20200032). A total of 16 male NZW rabbits were gained with half at 3 months old (young adult rabbits) and half at 12 months old (aged adult rabbits). The rabbits were anesthetized by injection of 3% w/v pentobarbital sodium via ear vein with a dose of 1 mL/kg. After extremities fixed, non-weight wearing trochlear ridge of the femur and tibial plateau were exposed by incising skin and muscle to create osteochondral defects below the subchondral bone plate and tibial defects connected with bone marrow (4 mm in diameter) in each hind legs of rabbits in order to allow bone marrow penetration into the constructs (Figure 1), respectively. The fibrin-based constructs were then implanted into the defects, followed by suturing muscle and skin with 3-0 non-absorbable surgical suture (Hangzhou Huawei Medical Instruments Co., Ltd., China). Previous studies have demonstrated that the osteochondral trauma with a size of 4 mm is critically large and cannot be repaired without implantation of constructs or scaffolds (Deng et al., 2017; Shi et al., 2017). To avoid unnecessary sacrifice of animals, the blank control was not set in this study. After the rabbits were injected with an overdose of sodium pentobarbital at 12 weeks post-surgery, two young adult rabbits and two aged adult rabbits were used to harvest the samples, obtaining four samples in every group for microcomputed tomography (μ-CT) analysis and histological analysis. At 18 weeks post-surgery, the remained rabbits were sacrificed to harvest three samples for the same characterizations and five samples stored at −80°C in each group.

### μ-CT Reconstruction and Histological Analysis

The obtained fresh samples were scanned by a μ-CT imaging system (μCT-100, SCANCO Medical AG, Switzerland) to evaluate the newly formed tissues and evaluate regenerative effects through two-dimensional (2D) reconstruction, three-dimensional (3D) reconstruction and quantitative analysis. After μ-CT analysis, the samples were fixed using 4% formaldehyde solution for about 2 weeks, and then demineralized by 10% ethylenediamine tetraacetic acid disodium salt dehydrate (EDTA⋅Na_2_) for at least 2 months. The samples were finally dehydrated by graded ethanol and embedded in paraffin. The paraffin sections were stained with hematoxylin and eosin (H&E), periodic acid Schiff (PAS), Safranine O and fast green for observation of extracellular matrix deposition, glycosaminoglycans (GAGs) deposition, and cartilage and bone, respectively, by Facility for Histomorphology, School of Medicine of Zhejiang University.

### Statistical Analysis

All data were expressed as mean ± standard deviation (SD, *n* = 3). The significant analysis was performed by *t*-test. A value of ^∗^*p* < 0.05 was regarded as significantly different.

## Results

The fibrinogen exists abundantly in serum, showing very good biocompatibility and enhancing cell–substrate interactions. It is readily degraded *in vivo* without the production of side products eliciting possible inflammatory responses as those from synthetic polyesters. In this study, the macroporous fibrin scaffold was prepared, into which fibrin gel, BMSCs, and lipofectamine/pDNA-TGF-β1 complexes were loaded to obtain the fibrin-based construct for the regeneration of osteochondral and tibial defects of both young and aged adult rabbits *in vivo*. The fibrin gel can offer a more biomimetic environment to support the proliferation and differentiation of BMSCs.

### Properties of the Fibrin-Based Scaffold

**FIGURE 2:**
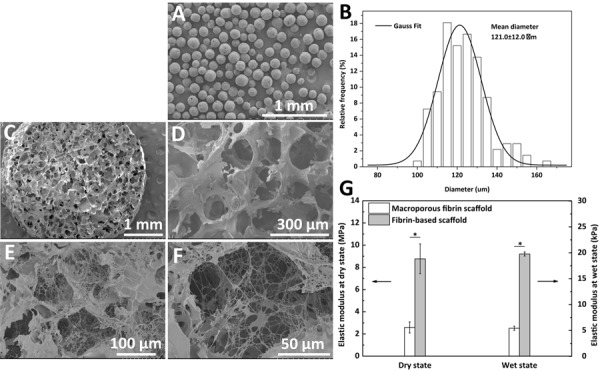
SEM images of **(A)** polycaprolactone (PCL) microspheres, **(C,D)** macroporous fibrin scaffold, and **(E,F)** fibrin-based scaffold. **(B)** Diameter distribution of PCL microspheres fitted according to Gauss formula, and **(G)** elastic moduli of the macroporus fibrin scaffold and fibrin-based scaffold. “*” represents that the compared data are statistically different at the significant level of 0.05.

The morphology of the PCL microspheres (A), macroporous fibrin scaffold (C,D), and fibrin-based scaffold (E,F) is shown in Figure 2. PCL microspheres (100–200 μm in diameter) exhibited a regular spherical structure with a mean diameter of 121.0 ± 12.0 μm according to statistical analysis (Figure 2B). An optimal PCL to fibrinogen mass ratio of 1.5: 1 was used to prepare the macroporous fibrin scaffold (Dai et al., 2016), which showed interconnected macro-pores (Figures 2C,D). After fibrin gel was introduced into the pores of the macroporous fibrin scaffold, fibrin fibers could be observed in the freeze-dried samples (Figures 2E,F), whose structure is similar to meshwork of fibrin in the contracted clot (Cines et al., 2014). The porosity of the macroporous fibrin scaffold was (88.1 ± 1.3)%. This would imply there is abundant space in the macroporous fibrin scaffold, which facilitates interactions between cells and the biomaterial, and further promotes tissue regeneration (Zhang et al., 2014).

The elastic modulus of the fibrin-based scaffold was significantly improved in both the dry and wet states (Figure 2G). Comparatively, the elastic modulus in the wet state decreased significantly from MPa level to kPa level. The macroporous fibrin scaffold and fibrin-based scaffold had an elastic modulus of 5.4 ± 0.4 and 19.8 ± 0.4 kPa at a wet state, respectively (Figure 2G). The enhanced mechanical property of this fibrin-based scaffold in comparison to macroporous fibrin scaffold would be beneficial to cartilage and bone regeneration.

The weight loss of the macroporous fibrin scaffold and fibrin-based scaffold was measured in 0.01 M PBS at 37°C under sterilized condition. Supplementary Figure S1 shows that the weight loss rates for the macroporous fibrin scaffold and fibrin-based scaffold were similar. About 45% weight was remained for both of them at 3 days. Furthermore, both types of scaffolds almost completely “disappeared” at 28 days.

### Cellular Compatibility and Effects of BMSCs Concentration on the Behavior of the Fibrin-Based Scaffold

The morphology and cytoviability of BMSCs within the fibrin-based scaffold were investigated. The CLSM images shows good adhesive and spreading behavior of BMSCs within the fibrin-based scaffold after being cultured for 24 h *in vitro* (Supplementary Figures S2A–E). In addition, BMSCs could proliferate significantly after 7 days *in vitro* incubation in the scaffold according to the MTT assay (Supplementary Figure S2F), demonstrating good cellular compatibility of the fibrin-based scaffold.

**FIGURE 3:**
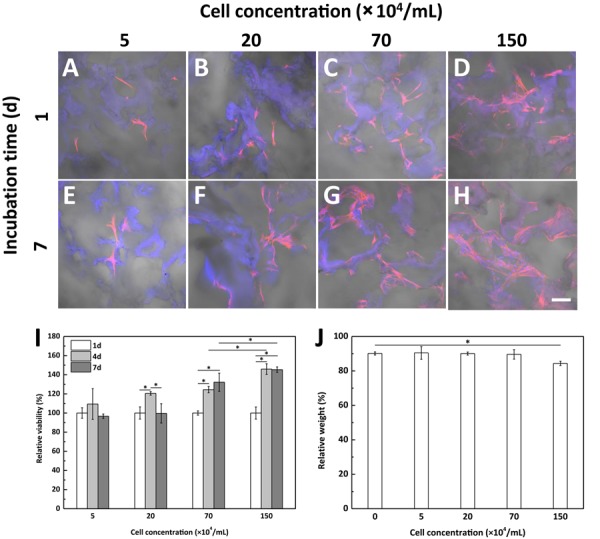
CLSM images of bone marrow-derived mesenchymal stem cells (BMSCs) with different seeding concentration within the fibrin-based scaffold after being incubated for **(A–D)** 1 day and **(E–H)** 7 days at 37°C and 5% CO_2_ atmosphere. Scale bar: 100 μm. BMSCs were loaded into the scaffold with a cell concentration of **(A,E)** 5.0 × 10^4^/mL, **(B,F)** 2.0 × 10^5^/mL, **(C,G)** 7.0 × 10^5^/mL, and **(D,H)** 1.5 × 10^6^/mL, respectively. Effects of cell concentration on **(I)** cell proliferation and **(J)** degradation behavior of the fibrin-based scaffold after being cultured *in vitro* for 7 days (*n* = 3). “*” represents that the compared data are statistically different at the significant level of 0.05.

When performing *in vitro* culture of BMSCs within the scaffold, despite of the good cellular compatibility of the scaffold, we found that this BMSCs-laden fibrin-based scaffold was degraded very fast at high cell concentration of 5.0 × 10^6^/mL. Therefore, we further studied the effects of cell concentration on cell behaviors and scaffold degradation. The spreading and distribution of BMSCs within the fibrin-based scaffolds were observed by CLSM (Figures 3A–H), showing the obvious cell proliferation at relatively higher cell concentrations (Figures 3C,D,G,F,I) after being incubated *in vitro* for 7 days. Concretely, BMSCs proliferated significantly after cultured for 4 and 7 days in the scaffold with an incorporating cell concentration of 7.0 × 10^5^/mL or 1.5 × 10^6^/mL (Figure 3I). By contrast, lower cell concentration did not contribute to cell proliferation obviously. In addition, after degradation, the relative weight of the fibrin-based scaffold incorporated with cells at lower concentrations (smaller than 7.0 × 10^5^/mL) was almost the same as that of acellular scaffold, whereas the cell concentration of 1.5 × 10^6^/mL would result in significantly lower relative weight after BMSCs were cultured *in vitro* for 7 days (Figure 3J). Therefore, further increase of incorporating cell concentration would accelerate degradation process of the fibrin-based scaffold. Taking these results into consideration, an incorporating cell concentration of 1.5 × 10^6^/mL was chosen for subsequent animal experiment.

### Evaluation on Repair Effect

For animal experiment, adult BMSCs isolated from bone marrow of 3 months old rabbits were loaded within the fibrin-based scaffold in combination with lipofectamine/pDNA-TGF-β1 complexes, which were implanted into osteochondral defects and tibial defects of both young and aged adult rabbits. It is reported that MSCs from younger donors could maintain better functions such as adhesion and proliferation behaviors and differentiation potential than those from older donors (Kretlow et al., 2008; Shen et al., 2011). Therefore, this operation is believed to be feasible and beneficial to aged adult rabbits.

**FIGURE 4:**
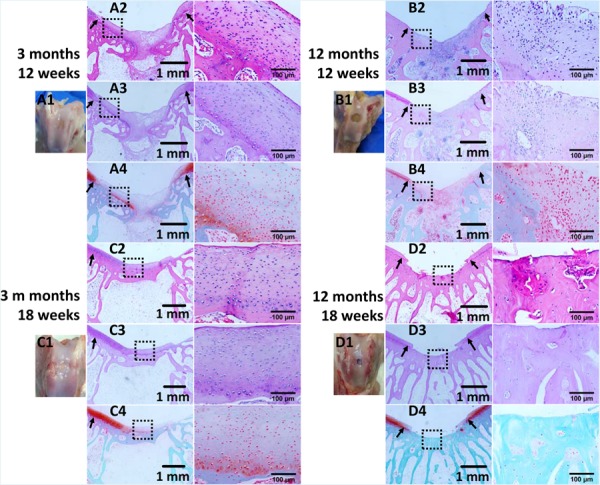
Histological analysis of osteochondral defects implanted with the fibrin-based construct at **(A,B)** 12 weeks and **(C,D)** 18 weeks post-surgery. **(A1–D1)** Gross view, **(A2–D2)** H&E staining for extracellular matrix (ECM) deposition, **(A3–D3)** PAS staining for glycosaminoglycans (GAGs), and **(A4–D4)** safranine O and fast green staining for GAGs and collagen. Black arrows indicate boundaries between normal tissues and neo tissues. The parts marked with black boxes are magnified in the right adjacent images, respectively. Rabbits for animal experiment were **(A,C)** 3 months old (young adult rabbits), and **(B,D)** 12 months old (aged adult rabbits) when the surgery was operated.

**FIGURE 5:**
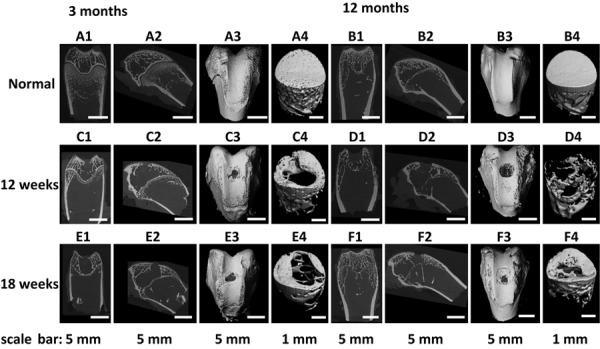
**(A1–F1)** Coronal plane and **(A2–F2)** sagittal plane μ-CT two-dimensional (2D) reconstruction images and **(A3–F3)** field of view, and **(A4–F4)** region of interest μ-CT three-dimensional (3D) reconstruction images of **(A,B)** normal osteochondral tissue, and **(C–F)** osteochondral defects implanted with the fibrin-based construct at **(C,D)** 12 weeks and **(E,F)** 18 weeks post-surgery. Rabbits for animal experiment were **(A,C,E)** 3 months old (young adult rabbits) and **(B,D,F)** 12 months old (aged adult rabbits) when the surgery was operated.

**FIGURE 6:**
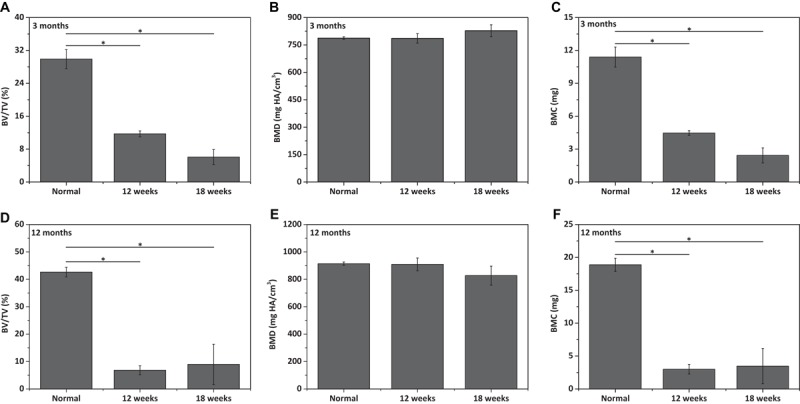
Qualitative analysis for **(A,D)** bone volume (BV)/total volume (TV), **(B,E)** bone mineral density (BMD), and **(C,F)** bone mineral content (BMC) of neo-subchondral bone tissues in **(A–C)** 3 months old rabbits and **(D–F)** 12 months old rabbits (*n* = 3). “*” represents that the compared data are statistically different at the significant level of 0.05.

The repair effect of cartilage and subchondral bone for both young and aged adult rabbits was evaluated by gross view and histological examination (Figure 4 and Supplementary Figures S3, S4), and μ-CT analysis (Figures 5, 6). The gross view shows one of four fibrin-based construct-implanted osteochondral defects was filled by neo-tissues with a similar color to the native hyaline cartilage for the young adult rabbits at 12 weeks post-surgery (Figure 4A1 and Supplementary Figure S3), and two of three at 18 weeks post-surgery (Figure 4C1 and Supplementary Figure S4). However, the defects for the aged adult rabbits still existed without obvious cartilage coverage at 12 weeks (Figure 4B1 and Supplementary Figure S3) and 18 weeks (Figure 4D1 and Supplementary Figure S4) post-surgery. Histological images further show that the neo-tissues were positively stained with some PAS and Safranine O (Figures 4A3,A4,C3,C4) for the young adult rabbits, indicating synthesis of hyaline cartilage-specific GAGs and the formation of developing neo-hyaline cartilage tissues. By contrast, there was no GAGs deposition in the aged adult rabbits (Figures 4B3,B4,D3,D4). It was also observed that the fibrin-based construct was totally degraded at 12 weeks post-surgery. In addition, the histological images indicate the repair of subchondral bone, which was further confirmed by μ-CT 2D and 3D reconstruction (Figure 5), and quantitative analysis (Figure 6). The μ-CT reconstruction images show that neo-subchondral bone tissues were developed in both young and aged adult rabbits with higher porosity (Figures 5C–F) compared with the normal subchondral bone (Figures 5A,B). According to μ-CT quantitative results, the normal subchondral bone of aged adults exhibits relatively a higher bone volume (BV)/total volume (TV), bone mineral density (BMD), and bone mineral content (BMC) (Figures 6D–F) compared with young adult rabbits (Figures 6A–C). Therefore, although similar BV/TV and BMC were found in both young and aged rabbits, the neo-subchondral bone tissues in young adult rabbits achieved a higher repair degree. All these results indicate that the osteochondral defects were partially repaired for the young adult rabbits, whereas only the subchondral bone of aged adult rabbits was partially restored.

**FIGURE 7:**
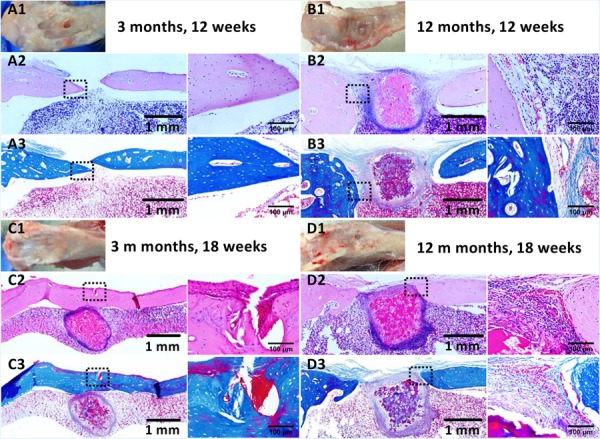
Histological analysis of tibial defects implanted with the fibrin-based construct at **(A,B)** 12 weeks and **(C,D)** 18 weeks post-surgery. **(A1–D1)** Gross view, **(A2–D2)** H&E staining for extracellular matrix (ECM) deposition, and **(A3–D3)** Masson staining for collagen. Black arrows indicate boundaries between normal tissues and neo tissues. The parts marked by the black boxes are magnified in the right adjacent images, respectively. Rabbits for animal experiment were **(A,C)** 3 months old (young adult rabbits) and **(B,D)** 12 months old (aged adult rabbits) when the surgery was operated.

**FIGURE 8:**
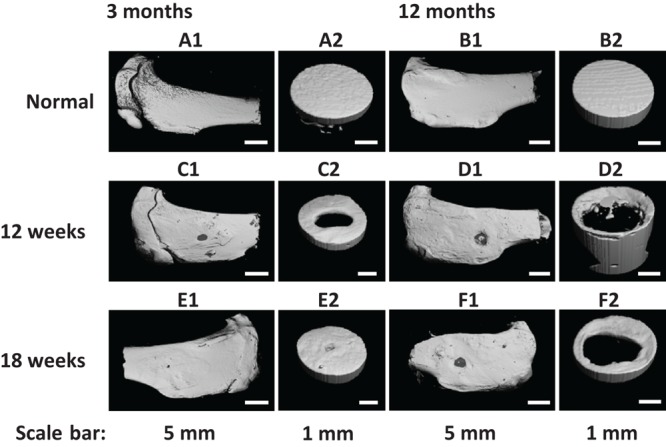
**(A1–F1)** Field of view and **(A2–F2)** region of interest μ-CT three-dimensional (3D) reconstruction images of **(A,B)** normal tibia and **(C–F)** tibial defects implanted with the fibrin-based construct at **(C,D)** 12 weeks and **(E,F)** 18 weeks post-surgery. Rabbits for animal experiment were **(A,C,E)** 3 months old (young adult rabbits) and **(B,D,F)** 12 months old (aged adult rabbits) when the surgery was operated.

**FIGURE 9:**
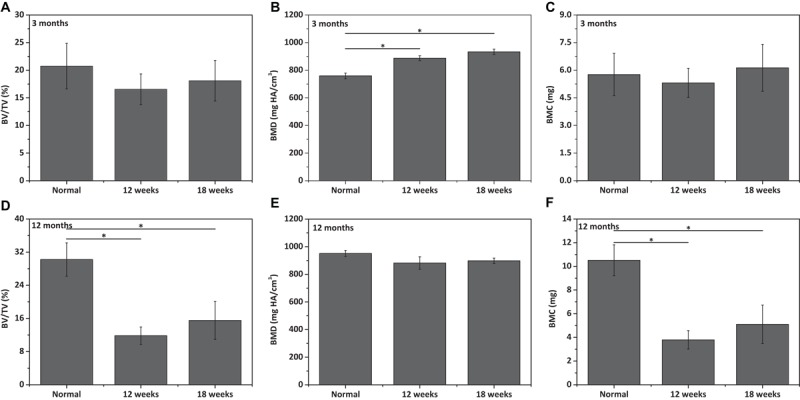
Qualitative analysis for **(A,D)** bone volume (BV)/total volume (TV), **(B,E)** bone mineral density (BMD), and **(C,F)** bone mineral content (BMC) of neo-tibial bone tissues in **(A–C)** 3 months old rabbits and **(D–F)** 12 months old rabbits (*n* = 3). “*” represents that the compared data are statistically different at the significant level of 0.05.

Similarly, the repair effect of tibial defects for both young and aged adult rabbits was assessed by gross view and histological analysis (Figure 7 and Supplementary Figures S5, S6), and μ-CT analysis (Figures 8, 9). According to the gross view and H&E images, four tibial defects implanted with the fibrin-based construct were obviously reduced (Figure 7A and Supplementary Figure S5) in the young adult rabbits at 12 weeks post-surgery. Moreover, all 3 defects were regenerated with complete closure at 18 weeks post-surgery (Figure 7C and Supplementary Figure S6). The defects implanted with the constructs were also decreased in the aged adult rabbits, but they were only partially repaired at 18 weeks post-surgery (Figures 7B,D and Supplementary Figures S5, S6). Masson staining was used to further estimate the neo-bone formation, indicating the repair process of the tibial defects in both young and aged adult rabbits (Figure 7). Collagen fibers are stained blue, and usually the darker of the color, the more mature of the neo-bone tissues. The neo-cortical bone exhibited similar morphology with the native bone without boundaries between them in the young and aged adult rabbits (Figures 7A3–D3). In some cases, induced inflammatory responses were observed within the defects with a few collagen fibers observed around the implanted fibrin-based constructs, which hinder the degradation of the constructs to some extent (Figures 7B2,B3,C2,C3,D2,D3). The cell types surrounding the implanted fibrin-based constructs were further analyzed. The H&E staining images reveal that numerous lymphocytes and neutrophils were accumulated toward the residual constructs (Supplementary Figure S7). Fibroblasts were also observed around the debris areas. The μ-CT 2D and 3D reconstruction images also show that cortical bone was formed within the tibial defects with full restoration in the young adult rabbits and partial healing in the aged adult rabbits, respectively (Figure 8). μ-CT quantitative analysis reveals that the aged adult rabbits possessed higher BV/TV, BMD, and BMC than those of young adult rabbits (Figure 9). Meanwhile, the regenerated tibial bone in the young adult rabbits exhibited similar BV/TV and BMC, and higher BMD compared with the native one, while the repaired bone in the aged adult rabbits presented relatively lower BV/TV and BMC with similar BMD achieved, further conforming the results of histological analysis and μ-CT reconstruction. Therefore, the fibrin-based constructs could realize almost complete regeneration of tibial defects at the young adult rabbits, whereas partial repair at the aged adult rabbits.

## Discussion

In this work, a fibrin-based construct was fabricated by using PCL microspheres as porogen for the preparation of the macroporous fibrin scaffold and a negative pressure method for loading of fibrin gel/BMSCs/(lipofectamine/pDNA-TGF-β1) complexes into the scaffold. It was then used to study the repair of osteochondral and tibial defects created in both young and aged adult rabbits. Fibrin, a natural biomaterial originated from the pre-protein fibrinogen, possesses outstanding biocompatibility, injectability, and biodegradability, and thus is usually used as tissue adhesives, carriers of tissue specific cells, and bioactive factors in drug delivery and regenerative medicine (Chen et al., 2013). However, the poor mechanical behavior of fibrin gel significantly limits its applications in cartilage and bone regeneration (Frenkel and Di Cesare, 2004; Basha et al., 2015). Here, we combined the suitable mechanical property of the macroporous fibrin scaffold with the delivery capability of the fibrin gel to facilitate the use of fibrin for cartilage and bone tissue engineering.

The macroporous fibrin scaffold with interconnected pores and sufficient porosity could allow cell penetration and migration, adequate exchange of nutrients for cell survival and proliferation, and further ingrowth of newly formed tissue even after fibrin gel filling (Song et al., 2018). In addition, the fibrin gel, together with the macroporous fibrin scaffold, could provide a suitable microenvironment for BMSCs. According to the results of compression test, the elastic modulus of the macroporous fibrin scaffold was significantly higher than that of fibrin gel (Snyder et al., 2014), and the loading of fibrin gel into the scaffold enhanced the mechanical behavior of the fibrin-based scaffold greatly. The fibrin gel plays a key role in improvement of the modulus of the fibrin-based scaffold. The fibrin gel could combine with the macroporous fibrin scaffold closely after thrombin crosslinking, and thereafter absorb and dissipate the compressive load imposed on the fibrin-based scaffold, which is similarly to the fiber reinforcement (Fantilli et al., 2017) and also proteins between the strong aragonite layers endowed with ductility and toughness (Wegst et al., 2015; Zhang et al., 2016). It is documented that BMSCs tend to differentiate into chondrocytes when the substrate modulus is at 1–100 kPa (Han et al., 2016), so an elastic modulus of 19.8 ± 0.4 kPa at the wet state of this fibrin-based scaffold would be beneficial to chondrogenic differentiation of BMSCs. In addition, BMSCs could differentiate into osteoblasts in a wide range of substrate elasticity (5 kPa–3 MPa) (Han et al., 2016). Therefore, this fibrin-based scaffold could support cartilage and bone regeneration from a perspective of the stiffness of biomaterials.

The weight loss of the macroporous fibrin scaffold was quite different from that incubated in complete DMEM under the same condition (Dai et al., 2016), which is associated with the structure and properties of fibrin crosslinked by thrombin. Fibrinogen is first cleaved by thrombin to yield fibrin monomers, which are then physically assembled by knob-hole interactions to form fibrin. This assembled fibrin could be further stabilized by chemically crosslinking using factor XIII, a kind of glutamyltransferase (Roberts et al., 2001; Litvinov et al., 2005). The fibrin crosslinked by thrombin might be unstable in PBS, leading to dissociation and dissolution. When immersed in DMEM, however, the macroporous fibrin scaffold could be stabilized by plasma protein existing in FBS to some extent. Therefore, it is speculated that this fibrin-based scaffold would have a relatively longer degradation period *in vivo* to provide sufficient mechanical support before tissue ingrowth.

The *in vitro* data suggest that BMSCs could adhere, spread, proliferate, and maintain their spindle-shaped morphology within the fibrin-based scaffold, confirming the good cytocompatibility of the scaffold. Meanwhile, the degradation of fibrin-based scaffold loaded with BMSCs exhibited a cell concentration-dependent manner due to the cell-mediated degradation feature of fibrin (Arulmoli et al., 2016). In consideration of influences of encapsulating concentration of BMSCs on cell proliferation and degradation rate of the fibrin-based construct, a cell concentration of 1.5 × 10^6^/mL was used for the animal experiment to provide a relatively low degradation rate to adapt to the repair process while ensuring proliferation of BMSCs within the scaffold.

On the basis of *in vitro* experiment, we implanted this fibrin-based construct laden with BMSCs and lipofectamine/pDNA-TGF-β1 complexes into osteochondral and tibial defects of young and aged adult rabbits to investigate the repair effects in their respective tissue-specific microenvironments. In this system, latent endogenous and transfected TGF-β1 by laden BMSCs and other tissue-resident cells exerts important roles on recruitment of MSCs/progenitor cells and inflammation-related cells, and regulation of cell fate to take part in tissue regeneration process after ligand activation in response to microenvironment perturbation (Xu et al., 2018). In addition, aging is usually accompanied by immune senescence and decline of interactions with macrophages, migration capability, and differentiation potential of BMSCs (Kovaiou et al., 2007; Lin et al., 2019), which should be taken into consideration when evaluating the repair effects.

According to the histological and μ-CT data, for the young adult rabbits, the osteochondral defects implanted with the fibrin-based constructs were partially repaired with developing cartilage formed in the cartilage layer and bone matrix deposition in the subchondral bone region. Compared with some reported *in vivo* results performed at osteochondral defects of the same size implanted with fibrin containing constructs, similar repair effects were achieved using the fibrin-based constructs at 12 weeks post-surgery (Vogt et al., 2009; Xie et al., 2012). For instance, hyaline cartilage-like neo tissues were developed within the osteochondral defects (4 mm in diameter) treated by a platelet-rich fibrin scaffold loaded with BMSCs (Xie et al., 2012). In addition, the repair effects in this work were not significantly improved in comparison with that of the acellular macro-porous fibrin scaffold implanted defects (Dai et al., 2016), which is likely caused by the relatively faster *in vitro* and *in vivo* degradation rates of the fibrin-based construct.

As proved by the H&E and Masson staining images, the tibial defects were covered by neo-cortical bone tissues with abundant bone matrix deposition and good integration with the adjacent native bone tissues after implantation of the fibrin-based constructs at the young adult rabbits for 12 and 18 weeks. The regeneration effects were further confirmed by similar BV/TV and BMC with the normal tibial tissues (Figures 9A,C). Allografts and synthetic bone substitutes are clinically used as bone substitutes to treat bone defects (Puppi et al., 2010; Huey et al., 2012). Human dentin grafts were applied to treat rabbit circular tibial defects (6 mm in diameter), resulting in perfect regeneration with replacement of the grafts by new bone after 3 and 6 months, while the blank control group was just partially repaired (Andersson et al., 2009; Andersson, 2010). Similar repair effects were achieved by using the fibrin-based constructs compared with the dentin grafts. Lee et al. (2010) fabricated bone matrix mimetic hydroxyapatite powder (HAP), hydroxyapatite cylinder (HAC), as well as hydroxyapatite-tri-calcium phosphate (HA-TCP) mixture cylinder to repair tibial defects with a diameter of 3.5 mm, which exhibited better repair effects than the titanium cylinder at 8 weeks post-surgery. Meanwhile, the BMD of the newly formed bone was smaller than that of the one repaired by the fibrin-based constructs and the normal tibiae of young adult rabbits (Lee et al., 2010). A Si-α tricalcium phosphate (Si-αTCP) scaffold was also prepared to treat tibial defects (6 mm in diameter), which achieved enhanced regeneration compared with the blank control group after implantation for 60 days (De Aza et al., 2017). The fibrin-based constructs showed superior repair effects compared with these artificial bioceramic scaffolds.

In our animal experiment models, osteochondral defects and tibial defects were perforated below the subchondral plate or connected with bone marrow allowing MSCs to penetrate into the constructs (Berthiaume et al., 2011). These MSCs, together with those from other adjacent niches involving cartilage, synovium, synovial fluid, joint adipose tissue, and periosteum, participate in the repair of osteochondral and tibial defects (McGonagle et al., 2017). However, with these endogenous reparative cells alone, functional or complete repair could not be realized. For instance, fibrous tissues or fibrocartilage was formed within those osteochondral defects without further treatment (Xie et al., 2012; Jang et al., 2013). The untreated tibial defects (3.5–6 mm in diameter) were partially repaired and filled with neo-bone tissues inferior to native ones (He et al., 2007; Andersson et al., 2009; Lee et al., 2010). By combing the endogenous reparative abilities with the fibrin-based construct, the repair effects of the injured tissues could be significantly improved as demonstrated by the *in situ* repair results. TGF-β1 also plays important roles during tissue remodeling processes. TGF-β1 recruits reparative cells and mediates the differentiation of MSCs under the assistance of other coexisted signals in specific microenvironments, and thus orchestrates the remodeling and repair of injured tissues (Xu et al., 2018). It is reported that a chitosan sponge scaffold modified with TGF-β1 affinity peptides and preloaded with TGF-β1 enhances *in vitro* chondrogenic differentiation of MSCs and *in vivo* ectopic cartilage formation and *in situ* cartilage regeneration (Chen et al., 2018). In our previous work, superior regeneration of full-thickness cartilage defects was realized by a BMSCs and TGF-β1 loaded PLGA/fibrin gel construct with upregulated expression of chondrogenic-related genes in neo-tissues compared with that of the TGF-β1 absent construct, indicating the important roles of TGF-β1 in facilitating chondrogenesis of BMSCs and cartilage regeneration (Wang et al., 2010b). Within bone microenvironments, TGF-β1 acts as a coupling factor which is activated in the bone resorptive sites and subsequently directs the migration of BMSCs to initiate bone formation in response to local signals (Tang et al., 2009). Therefore, it is believed that the *in situ* secreted TGF-β1 within the fibrin-based construct and endogenous reparative cells would contribute to the repair of osteochondral and tibial defects.

The histological images also reveal different degradation rates of the implanted fibrin-based constructs in the osteochondral and tibial microenvironments. The degradation dynamics of the constructs did not exhibit an age-dependent manner. In the region of osteochondral microenvironment, the fibrin-based constructs were degraded at 12 weeks post-surgery (Figure 4). However, in the region of tibial microenvironment, the construct debris still existed in bone marrow areas even at 18 weeks post-surgery. Therefore, the implanted constructs presented significantly slower degradation rate in tibial bone marrow than those in other concerned microenvironments. It is reported that the *in vivo* degradation of implanted biomaterials is regulated by innate tissue-specific immune cells and induced adaptive immune cells (Reid et al., 2015). Therefore, the host responses are defined by the specific tissue microenvironments, and thus influence the degradation kinetics of biomaterials (Reid et al., 2015). In the tibial bone marrow, mild chronic immune responses were produced with innate neutrophils and adaptive lymphocytes accumulated around the implanted fibrin-based constructs, but macrophage fused foreign body giant cells were not found on the interfaces of the constructs, indicating no foreign body reactions toward blood-derived fibrin (Chung et al., 2017).

For the aged rabbits, the subchondral bone and tibial bone were partially healed at 18 weeks after implantation of the fibrin-based construct, while the cartilage layer was not repaired absolutely. In addition, compared with those in the aged rabbits, a higher regeneration degree was achieved with regard to the repair of subchondral bone and tibial bone in the young adult rabbits. For the reasons of the obvious difference between young and aged adult rabbits referring to repair effects of cartilage, there are two aspects should be considered. On one hand, the functions of endogenous BMSCs decrease with age, including declined responsiveness to physical and injured environmental signals, and reduced homing or migration capacity and proliferation as well as chondrogenic abilities *in vivo* (Boyette and Tuan, 2014; Baker et al., 2015; Lin et al., 2019). It is also reported that BMSCs exhibit age-related characteristics of declined proliferation and chondrogenic potential *in vitro* (Zheng et al., 2007; Kretlow et al., 2008; Beane et al., 2014). On the other hand, age-related chondrocyte senescence causes increase of oxidative stress/damage and level of MMP-13 and pro-inflammatory cytokines such as IL-1, and decline of cell responses to growth factors such as TGF-β, basic fibroblast growth factor (bFGF), and insulin-like growth factor (IGF)-1 as well as their synthesis (Loeser, 2009; Rahmati et al., 2017). This change induces imbalance between catabolic activity and anabolic activity, and further alters biomechanical behaviors and induces cartilage matrix degradation, finally impairing articular cartilage homeostasis and remodeling capability (Loeser, 2009; Rahmati et al., 2017). In short, age-related dysfunction of MSCs and chondrocytes contributes to the repair effect differences.

With regard to subchonral bone and tibial bone regeneration, age-related alterations in bone marrow niches and differentiation potential of BMSCs could contribute to the relatively poor repair effect at the aged adult rabbits. It has been demonstrated that aging is usually related with delayed bone remodeling and formation as well as delayed bone marrow reconstruction in immunocompromised rats (Fisher et al., 2010). The increased level of ROS and/or energy metabolic defect during aging upregulates adipogenic-related transcriptional factors such as peroxisome proliferator-activated receptor-gamma (PPAR-γ) and downregulates osteogenic-related factors such as runt related transcription factor 2 (RUNX2), which shifts the differentiation potential of BMSCs from osteogenic to adipogenic lineage and accumulation of adipose tissue in bone marrow in long bones, resulting in reduced bone formation and increased susceptibility to bone fracture (Kim et al., 2012; Singh et al., 2016; Ambrosi et al., 2017). For all that, the subchondral bone and tibial bone were partially repaired by the fabricated fibrin-based construct, validating the positive role of this construct in the tissue remodeling and repair processes of bone tissues in the aged adult rabbits.

## Conclusion

A macroporous fibrin scaffold was fabricated by using PCL microspheres as porogen, into which fibrin gel was filled by a negative pressure method and thrombin crosslinking to obtain a fibrin-based scaffold. The porous structure of the scaffold with high porosity facilitated the interactions between cells and the material and nutrient exchange *in vitro* and *in vivo*. The suitable mechanical behavior of the fibrin-based scaffold could provide a proper microenvironment for exogenous and endogenous BMSCs to differentiate into targeted cells. The results of *in vitro* culture of BMSCs suggest the good biocompatibility of the scaffold and elucidate the influences of cell concentration on cellular viability and degradation behavior. The *in vivo* repair experiment of the fibrin-based construct found the partial repair of osteochondral defects and perfect regeneration of tibial defects in the young adult rabbits, and partial repair of subchondral bone and tibial bone defects in the aged adult rabbits. Comparatively, the same constructs achieved a higher repair degree of subchondral bone and tibial bone for the young individuals. These *in vivo* data may demonstrate the adaptability of the fibrin-based construct to particular microenvironments by tissue–material interactions. Further study should be performed to optimize the material system to achieve improved regeneration, especially at aged individuals, which would significantly benefit to clinical medicine.

## Data Availability Statement

The datasets generated for this study are available on request to the corresponding author.

## Ethics Statement

The animal study was reviewed and approved by the Animal Ethics Committee of Hangzhou Medical College.

## Author Contributions

XF, JY, and CG contributed the design of the study. XF carried out the experiments, data collection and analysis, and wrote the manuscript. XF, PX, TS, and YZ performed the animal experiments coordinately. All authors contributed to manuscript reading and revision and approved the submission.

## Conflict of Interest

The authors declare that the research was conducted in the absence of any commercial or financial relationships that could be construed as a potential conflict of interest.
