# Supplementary material for: Age-Related Regeneration of Osteochondral and Tibial Defects by a Fibrin-Based Construct in vivo
Source: Front Bioeng Biotechnol. 2020 May 5;8:404. doi: 10.3389/fbioe.2020.00404 (PMC7214756; doi:10.3389/fbioe.2020.00404)
Supplement: Supplementary file 1 [file Data_Sheet_1.pdf]

Supporting Information

Age-related regeneration of osteochondral and tibial defects by a fibrin-based construct *in vivo*

Xue Feng[1], Peifang Xu[2], Tao Shen[1], Yihan Zhang[1], Juan Ye[2*], and Changyou Gao[1*]

[1]MOE Key Laboratory of Macromolecular Synthesis and Functionalization, Department of Polymer Science and Engineering, Zhejiang University, Hangzhou 310027, PR China

[2]Department of Ophthalmology, the Second Affiliated Hospital of Zhejiang University, College of Medicine, Hangzhou, 310009, PR China

*** Correspondence:**
Changyou Gao
cygao@zju.edu.cn
Juan Ye
yejuan@zju.edu.cn

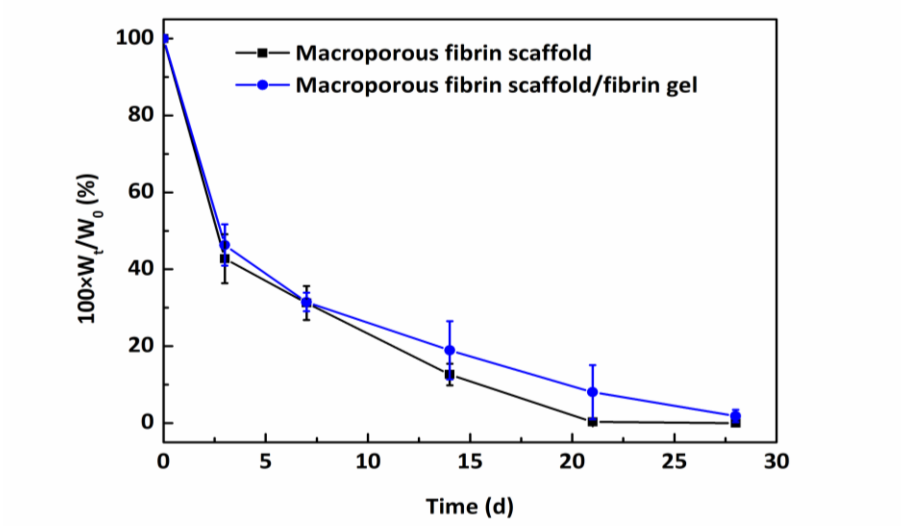

Fig. S1. Relative weight versus time of the macroporous fibrin scaffold and fibrin-based scaffold in 0.01 M PBS at 37 °C under sterilized condition (n=3).

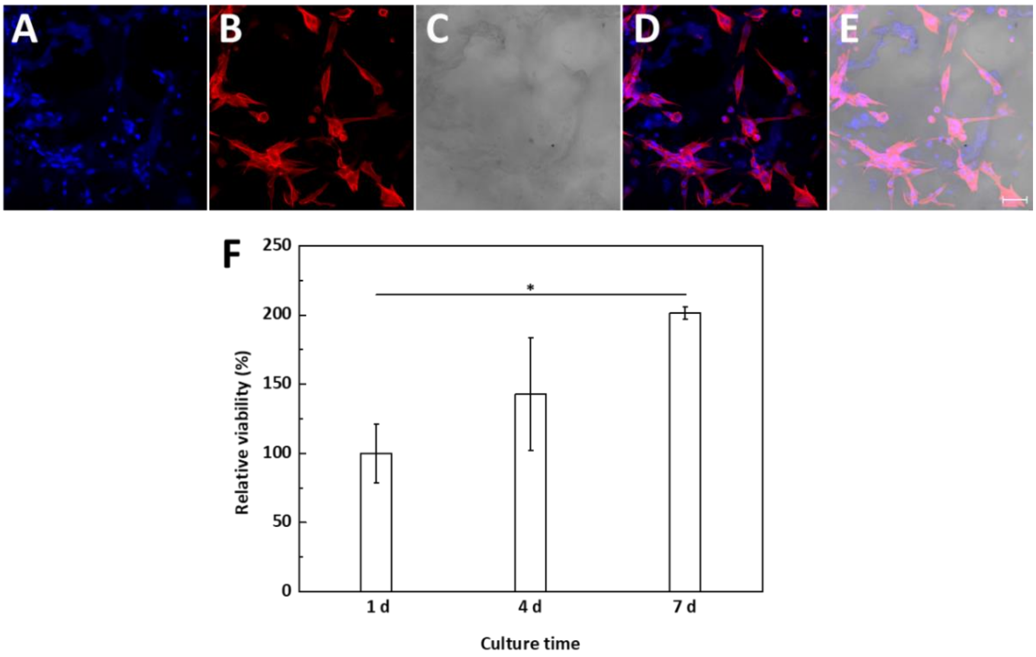

Fig. S2. (A-E) CLSM images of bone marrow-derived mesenchymal stem cells (BMSCs) within the fibrin-based scaffold being cultured for 24 h at 37 °C and 5% CO_2 atmosphere. (A) Nucleus stained by 2-(4-amidinophenyl)-6-indolecarbamidine dihydrochloride (4',6-diamidino-2-phenylindole) (DAPI, blue color), (B) cytoskeleton stained by rhodamine-labeled phalloidin (red color), (C) bright field, (D) merged image of A and B, and (E) merged image of (A-C). Scale bar: 50 µm. (F) Cytoviability assayed by MTT in the fibrin-based scaffold.

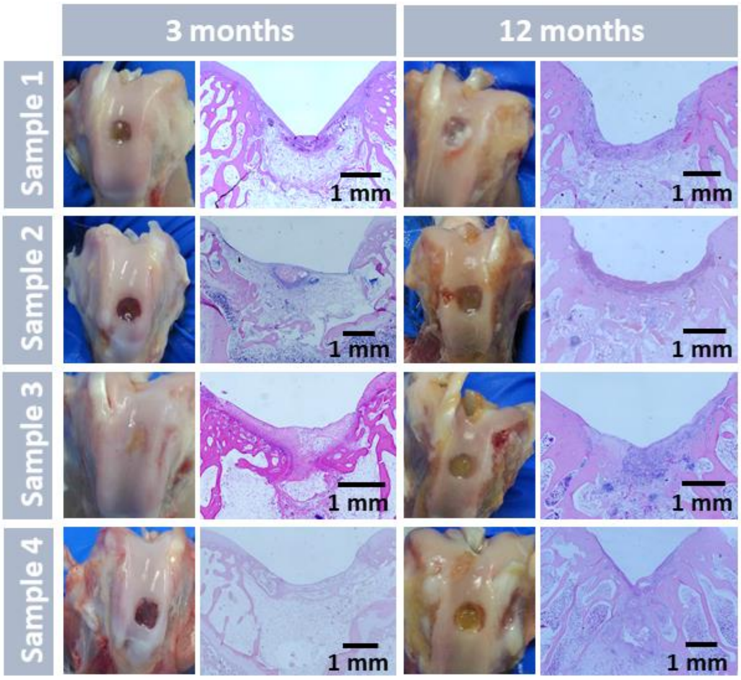

Fig. S3. Repair effects of osteochondral defects in young adult rabbits (3 months old) and aged adult rabbits (12 months old) at 12 weeks post-surgery evaluated by H&E staining.

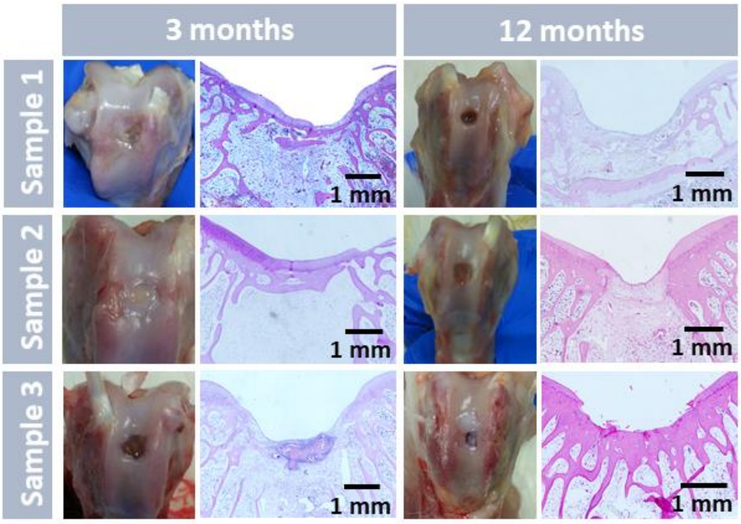

Fig. S4. Repair effects of osteochondral defects in young adult rabbits (3 months old) and aged adult rabbits (12 months old) at 18 weeks post-surgery evaluated by H&E staining.

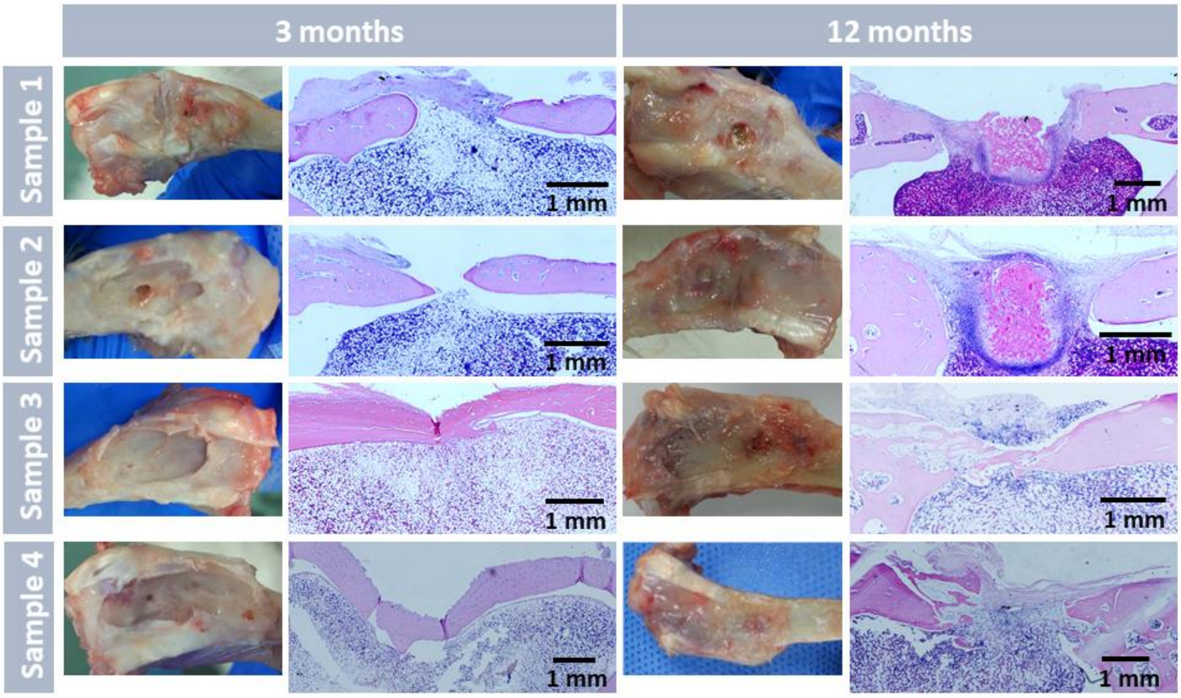

Fig. S5. Repair effects of tibial defects in young adult rabbits (3 months old) and aged adult rabbits (12 months old) at 12 weeks post-surgery evaluated by H&E staining.

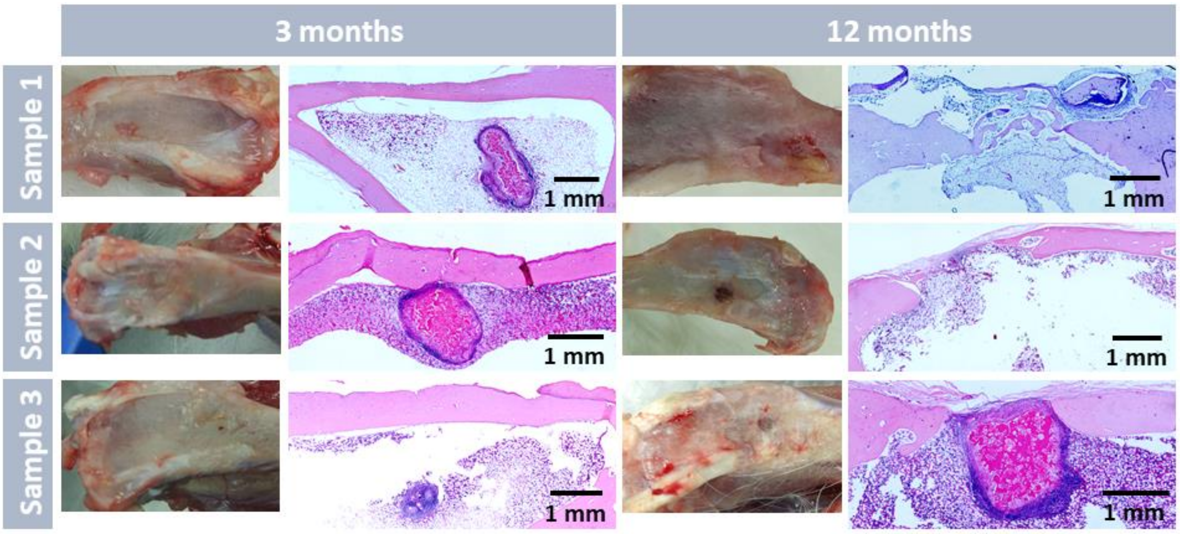

Fig. S6. Repair effects of tibial defects in young adult rabbits (3 months old) and aged adult rabbits (12 months old) at 18 weeks post-surgery evaluated by H&E staining.

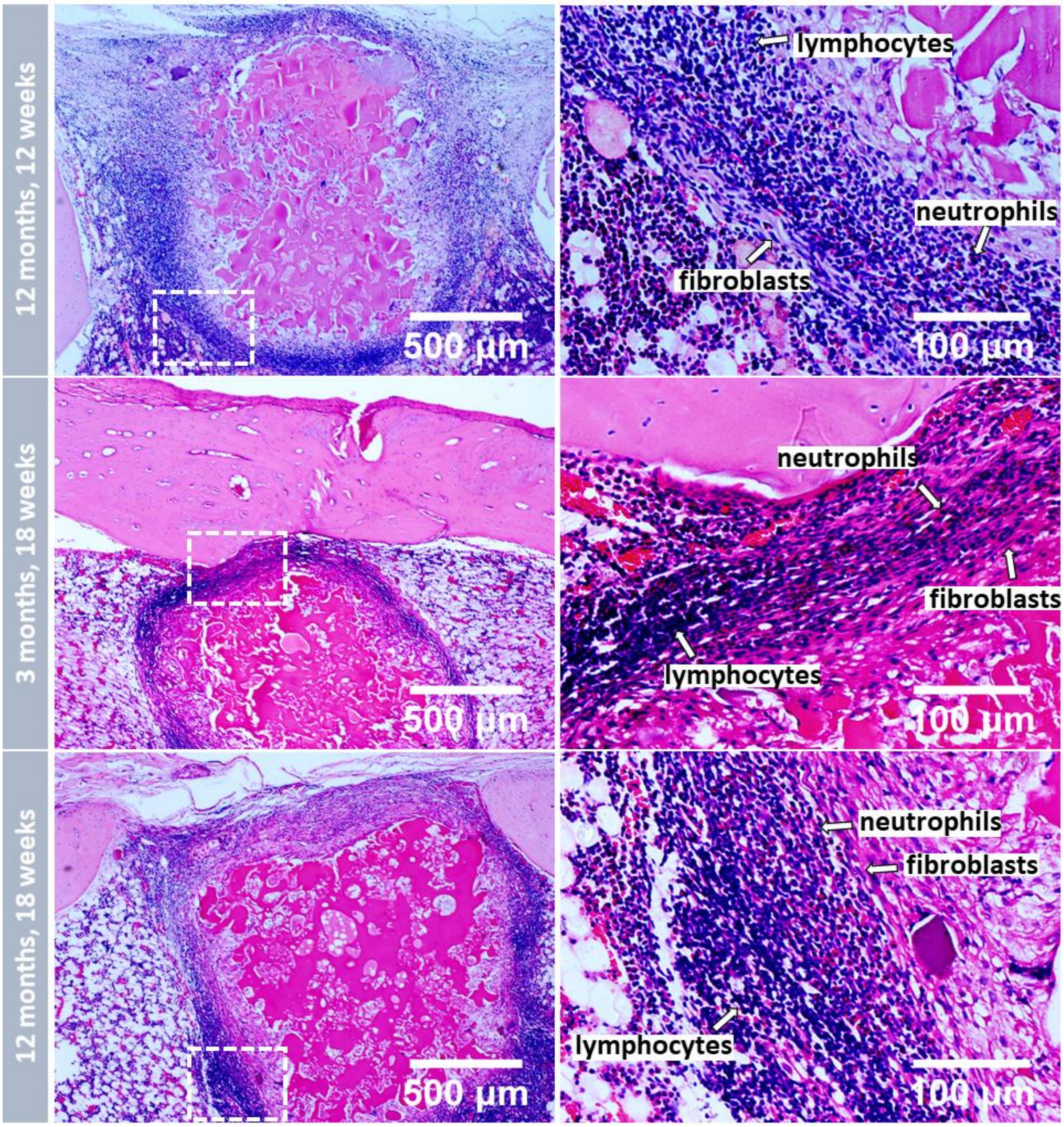

Fig. S7. Cell types surrounding the implanted fibrin-based constructs in tibial defects by H&E staining. Regions marked with white boxes are magnified in the right adjacent images.
